# A self-supervised machine learning pipeline for extracting information from live cell images at multiple doses and timepoints

**DOI:** 10.1038/s41598-025-32685-5

**Published:** 2026-01-07

**Authors:** Dmitry Yu. Isaev, Wen Pei Liu, Marc Cuevas, Yubo Tang, Kenny Ang, Chris Wilson, Quynh Mai, Jovani Adra, Allan Cruz, Khoi Nguyen, Michael Ricasa, Ankur Gupta, Mehrdad Hamadani, Deepa Sridharan, Yerem Yeghiazarians, Kurosh Ameri

**Affiliations:** Soley Therapeutics Inc., South San Francisco, USA

**Keywords:** Biological techniques, Computational biology and bioinformatics

## Abstract

Live cells are complex information-processing systems that continuously sense their environment and respond dynamically. However, conventional endpoint assays typically require fixation or cell destruction and fail to capture complex temporal changes. Live brightfield imaging offers a scalable, label-free solution that remains underutilized due to particularly low contrast, acute technical batch sensitivity, and the limited availability of robust computational methods for this modality. Leveraging recent self-supervised learning developments, we introduce Live Cell Dynamics (LCD), a novel end-to-end transformer-based pipeline, using novel plane-agnostic augmentation (treating different focal planes as views of the same state) and incorporating cross-batch sampling. LCD addresses brightfield modality challenges and extracts subtle dose- and time-dependent live cell states. Through systematic ablation we evaluate each self-supervised training innovation on a single cell line, measuring phenotypic activity (mean Average Precision) and Mechanism of Action (MoA) classification (F1-score), with 189 compounds in pre-training and 81 in holdout spanning ten MoAs. Our approach outperforms ablated baselines across all doses and timepoints for activity and MoA classification, enables compound polypharmacology detection from multi-dose/timepoint profiles, and supports unsupervised nuclei detection and counting. It leads to training foundation models from continuous live brightfield imaging to detect subtle live cell state changes, enabling scalable, cost-effective drug development.

**Supplementary Information:**

The online version contains supplementary material available at 10.1038/s41598-025-32685-5.

## Introduction

Live cells are complex information-processing systems that continuously sense their environment and respond dynamically via intricate mechanistic changes. An instantaneous configuration of cell biophysical responses, e.g. to a drug perturbation, constitutes a **cell state**—a latent multivariate physicochemical condition that describes how a cell will sense, interpret, and adapt to stimuli^1,2^. Modern drug discovery relies primarily on static endpoint assays that are in fact an observation of cell state at a frozen window in time, or on labeling techniques that can be dynamic yet are inherently biased through selection of reporter tags, e.g. Cell Painting images encoding morphology^3^, FRET traces capturing signaling flux of molecular events^4^, single-cell RNA-seq reporting transcriptional programs^5,6^, and proteomics quantifying protein abundance and modifications^7^.

These standard assays encode a broad range of biologically meaningful signals at different levels ranging from morphological and molecular changes. These signals in turn provide the opportunity for mechanistic interrogation of perturbations, especially when combined with handcrafted features that are typically more interpretable than deep-learning based models. However, these methods are not without limitations. For example, readouts can be limited to snapshot views of cell state at pre-selected doses and timepoints, due to the need for cell destruction (e.g. lysis, fixation), and/or fluorescent labeling (e.g. FRET biosensors, fluorescent stains). To capture dose-time dynamic changes, computational methods such as pseudo-temporal algorithms are developed to reconstruct “pseudo-trajectories” by analyzing many cells fixed at the same moment but existing in different states^8–10^.

Live cell brightfield microscopy serves as an affordable and scalable alternative that allows for nondestructive and continuous measurement of cellular dynamics. Compared to fluorescence imaging based on molecular markers, feature extraction from live brightfield images is challenging due to the low contrast^11^, heightened sensitivity to technical batch effects^12^, and higher visibility of natural cell phenotypic variance. In addition, without signals from biomarkers in brightfield microscopy, mechanistic interpretability remains challenging. Related work on brightfield datasets and models addresses some of these challenges to facilitate the widespread adoption of brightfield cell imaging. For example, LIVECell was developed as a public phase-contrast image dataset, even though it necessitates manual annotations^13^. More recently, the foundation model for image segmentation, SAM (Segment Anything), was finetuned for microscopy to segment cell images^14,15^; however, to further leverage brightfield imaging for downstream classification tasks such as activity detection, a feature extractor like CellProfiler is still needed to quantify the segmented morphology.

Here we develop a self-supervised foundation model as an end-to-end feature extractor for live brightfield microscopy without any manual annotations. We leverage recent advances in self-supervised learning^16,17^, a scalable approach capable of extracting meaningful patterns without manual labeling, to overcome the forementioned challenges. Self-supervised methods have already demonstrated success in analyzing various imaging data^18–22^, including fluorescence microscopy^23–33^, and combinations of label-free and fluorescent techniques^29,34,35^. A recent work by Forsgren et al.^36^ explored application of a self-supervised model pre-trained on RGB images to a limited set of seventy-one compounds from 6 MoAs, demonstrating promise of this approach, yet the work did not tackle the challenge of pre-training self-supervised models solely from live brightfield data modality.

**Fig. 1:**
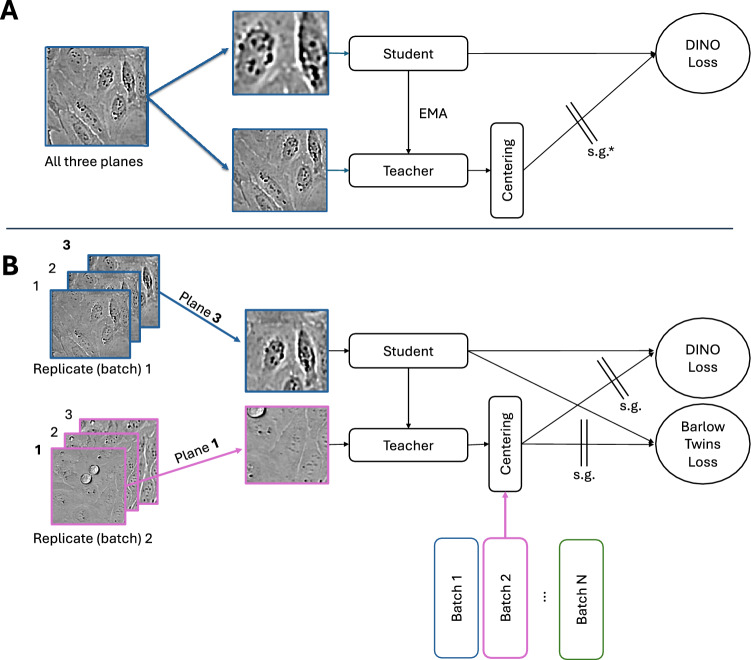
(**A**) Standard DINO framework. (**B**) Our model architecture, following the DINO framework and incorporating the cross-batch centering and Barlow Twins Loss enhancements proposed by Haslum et al. During augmentation, each branch (student and teacher) receives an image taken from a randomly chosen experimental replica (batch) and a randomly selected focal plane among the three available. The model maintains separate center vectors for each batch, which are applied during the teacher centering step based on the batch that is passed to the teacher branch. Training proceeds with two equally weighted loss functions—DINO and Barlow Twins—and our experiments reveal that cross-batch training is critical for learning semantically meaningful transformer attention structures (Fig. 4). *s.g.—stop gradient operation.

Our work addresses challenges of brightfield modality through a novel pipeline we call **Live Cell Dynamics (LCD) to enable label-free and imaging-based phenotypic profiling**. It consists of a foundation ViT model as a feature extractor, together with the downstream metric and classifier to detect compound activity and for MoA prediction. Notably, the ViT embeddings are compatible with a broad range of downstream tasks, and the pipeline design is inspired by latest advances in self-supervised learning models. Recent work has shown great promise to train foundation models for extracting biologically meaningful features without any manual annotation, and performance surpassing conventional methods such as CellProfiler has been reported in CellPainting datasets^23,26,37^. In this work, we leverage the DINO^18^ self-supervised training paradigm, modifying the training scheme with cross-batch sampling, Barlow Twins loss, and focal variation in brightfield imaging through novel plane-agnostic augmentation (Fig. 1). Our model architecture is specifically designed to address the challenges associated with self-supervised training using label-free, live brightfield microscopy. As previously described, brightfield imaging inherently suffers from a signal-to-noise ratio lower than other modalities such as fluorescence imaging^11,12^, typically by a few orders of magnitude. To overcome this challenge and to better distinguish biological variations from technical batch effects in brightfield microscopy, as described in Fig. 1, we implemented a cross-batch sampling method^38^ for live brightfield modality; we show this method encourages batch invariance for improved learning of biological effects, and indeed is an essential part of the model architecture for training foundation models without supervision. In addition, for each z-stack we randomly and uniformly sample from all planes, eliminating the need for artificial blur such as Gaussian blur in standard DINO^18^. Lastly, Barlow Twins loss was employed to encourage feature decorrelation^22^, and their respective contribution to the model performance is described in an ablation study.

With the pre-trained model as a backbone, we provide a live cell state recognition pipeline including feature extraction, normalization, phenotypic activity detection, and mechanism of action (MoA) classification. Importantly, key steps in the post-train pipeline, including the normalization and phenotypic activity detection metrics, are also designed to further mitigate the technical batch effects associated with brightfield imaging. Utilizing live brightfield scalability, we propose innovations in the normalization approach, for each FOV instance; this method was shown to alleviate technical effects at different levels including FOV and plate level, and to improve phenotypic activity detection. For activity detection metric we build upon the well-established mean average precision (mAP) framework^39^, and we propose the novel mAP effect size (mAP-ES) metric that incorporates null-distribution of the mAP over negative controls for estimating activity. The mAP-ES metric accounts for the strong technical effects observed in brightfield imaging, since technical effects are captured by the negative control distribution. Through systematic ablation we evaluate each self-supervised training method across all doses and timepoints on a single cell line treated with compounds—189 compounds (training) and 81 compounds (holdout)—spanning 10 mechanisms of action (MoAs), measuring phenotypic activity (mean Average Precision), and MoA classification (F1-score).

Leveraging flexibility of our foundation model, we further showcase its utilization in unsupervised live cell nuclei detection, which in turn provides a means for cell count without stains. When compared to cell count, our results indicate that our phenotypic activity pipeline is sensitive enough to detect subtle early phenotypic changes, long before visually interpretable changes, such as cell-stress states or death become evident. Furthermore, it improves MoA evaluations by incorporating time and dose dependent observations. With LCD, for the first time, we demonstrate the ability to reveal polypharmacology—indicating multiple mechanisms of action of the same drug—solely from live brightfield modality, relying on a comprehensive, dose-time specific map of live cell phenotypes.

In summary, we propose the first foundation model for live brightfield microscopy enabling a range of downstream tasks. Contributions of our current work are the following: (1) Novel pipeline architecture designed for live brightfield microscopy known to suffer from strong technical effects. Our pipeline adapts previously developed cross-batch sampling and Barlow Twins loss^38^, and introduces plane-agnostic augmentation during pre-train, and normalization and mAP-ES metric in the post-train pipeline. (2) Training on one of the largest live brightfield microscopy dataset (multi-replicate, multi-timepoint, 430K raw images in total), and evaluating on multi-replicate, multi-dose, multi-timepoint dataset (307K raw images from pre-train compound set, and 122K raw images from hold-out compound set). The hold-out dataset is shared with the broader community for future model development and evaluation. (3) Leveraging above, we demonstrate its power in a range of downstream tasks including activity detection, MoA prediction, poly-pharmacology characterization, nuclei detection, and temporal phenotypic trajectory tracking. As a universal feature extractor, our LCD model serves as a foundation for sensitive, accurate and cost-effective imaging-based profiling for drug discovery in the future.

## Results

We performed ablation studies to assess the contribution of augmentation and loss functions for self-supervised training, and different normalization methods following embedding extraction. For Phenotypic Activity Detection and MoA Classification, we systematically assessed plane-agnostic augmentation and each loss component’s contribution, conducting an ablation study across five model variants:

- Standard DINO loss (DINO)
- DINO + Barlow loss (DINO + Barlow)
- DINO + Cross-Batch loss (DINO + XB)
- DINO + Barlow + Cross-Batch loss (DINO + Barlow + XB)
- Plane-Agnostic Augmentation + DINO + Barlow + Cross-Batch loss (PA + DINO + Barlow + XB)

Each model was trained for 60 epochs on the same pre-training dataset further evaluated with attention maps. We confirm in live brightfield, the benefit of cross-batch sampling with both DINO and Barlow Twins losses to enable feature decorrelation, previously shown in self-supervised methods for fluorescent microscopy^38^. See Fig. 1 for the model architecture.

### LCD outperforms baselines in phenotypic activity detection

**Fig. 2:**
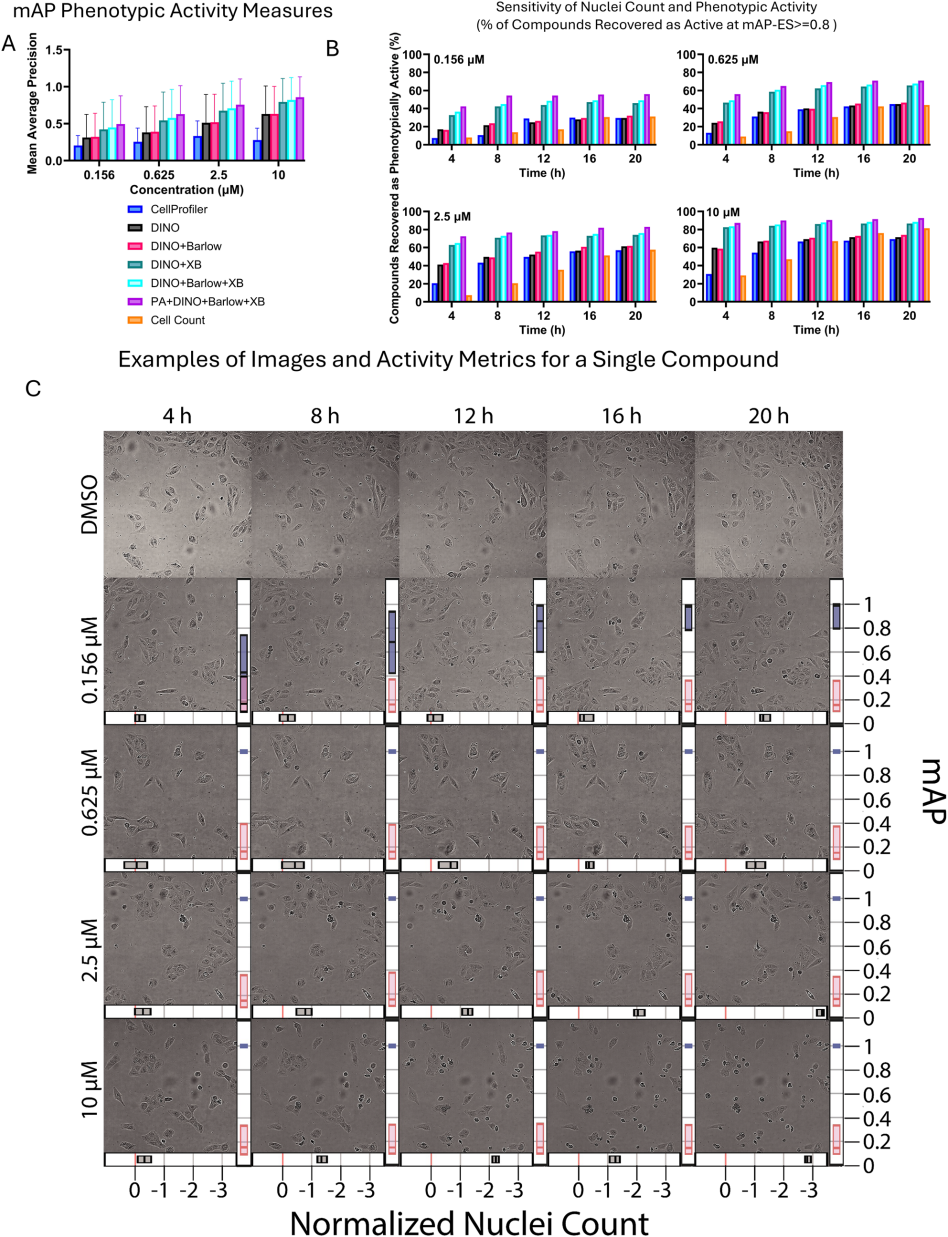
(**A**) Mean Average Precision (mean, standard deviation) for best performing normalization method (MAD + Harmony/FOV) across different pre-training approaches and CellProfiler, per each dose. A steady increase in phenotypic activity is observed as dose increases. Inclusion of cross-batch training substantially improves performance, with plane-agnostic training adding performance gains. (**B**) Sensitivity at mAP-ES threshold of 0.8 per timepoint for different pre-training approaches across doses at best performing normalization method (MAD + Harmony/FOV) for the pretraining set. Cell count baseline presented in orange. (**C**) Microscopy images of TAK-901, a polypharmacologic Aurora Kinase Inhibitor, at a range of doses and timepoints. Representative DMSO images at the corresponding timepoints are shown in the top row. Right inset of images: mAP 95% confidence interval for this compound (blue) and null distribution (red) at each dose and timepoint. Bottom inset: 95% confidence interval for median of the normalized live nuclei count (z-transformed DMSO mean is 0 noted by the red line). It is visible that in the early timepoints of the lowest doses, CI for median of normalized nuclei count overlaps with the DMSO mean, while mAP confidence intervals of compound and null distribution do not overlap in all but the lowest dose—earliest timepoint combination, indicating phenotypic activity even when the difference in cell count is not statistically significant.

On the pre-training dataset, all models had a steady mAP increase from 4 to 20 h. Our plane-agnostic ViT demonstrated highest mAP across models (Table 1 showing different normalization methods, and Fig. 2A, B showing model with best normalization method and varied training approaches). mAP performance between all pairs of models was significantly different, as measured by Wilcoxon paired signed-rank test. mAP performance between MAD + Harmony/FOV and all other normalization methods was significantly different. All pairwise comparisons are provided in Supplemental Materials (Tables SM3 and SM4). Comparison of different normalization methods demonstrated that our novel normalization method MAD + Harmony/FOV is the strategy resulting in highest mAP (Table 1).

**Table 1:** Mean Average Precision averaged across all doses and timepoints, per different pre-training approaches and normalization methods. Plane-agnostic training performs best across models, and MAD + Harmony/FOV provides the best performance across all normalization methods.

Model, training loss, augmentation	Normalization method mAP mean (std)			
	MAD	fastMNN	MAD + Harmony	MAD + Harmony/FOV
DINO	0.43 (0.37)	0.44 (0.36)	0.43 (0.37)	0.45 (0.37)
DINO + Barlow	0.44 (0.37)	0.44 (0.37)	0.44 (0.37)	0.46 (0.37)
DINO + XB	0.57 (0.39)	0.58 (0.38)	0.57 (0.38)	0.61 (0.38)
DINO + Barlow + XB	0.61 (0.39)	0.61 (0.38)	0.60 (0.39)	0.64 (0.38)
PA + DINO + Barlow + XB	0.66 (0.38)	0.67 (0.38)	0.66 (0.38)	**0.69 (0.37)**

We assessed the sensitivity of different methods to phenotypic activity across a broad range of mAP-ES thresholds (Fig. SM2). For model comparison at a single threshold and the downstream evaluations, we adopted a conventional threshold of effect size of 0.8 (Fig. 2B)^40^. Our results show that PA + DINO + Barlow + XB method consistently outperforms the baselines across the entire range of thresholds (Fig. SM2), indicating superior phenotypic response detection.

### Phenotypic activity detection outperforms cell count baselines

Cell count is a recently proposed baseline for assessing the added value of phenotypic profiling, as many activity benchmarks correlate strongly to viability, and can be predicted from a single cell count feature^41^. Live cell count per field of view was extracted for each image using a nuclei detection algorithm (see Methods for details). For each dose and timepoint, comparison of activity based on nuclei count with activity based on mAP-ES is shown in Fig. 2B and Fig. SM2. mAP-ES is consistently more sensitive to bioactivity than cell count alone, with the most pronounced differences in the lower doses and early timepoints. In Fig. 2C, a single compound is shown, with null and treatment mAP distributions, showing that mAP distributions allow to capture phenotypic activity even at very early dose, where nuclei count is not significantly different from the DMSO nuclei count. Additional analysis of sensitivity across mAP-ES thresholds and distribution-based definition of activity are presented in Supplemental Materials, section “Distributional Analysis of Activity”, and Figs. SM2 and SM3.

### Phenotypic activity detection outperforms CellProfiler single cell-level feature extraction baseline

Foundation models specifically for cell segmentation from microscopy images, demonstrated tremendous progress in recent years^14,15,42^. The most recent model, CellPose-SAM visually demonstrated strong performance for segmenting single U2OS cells from live brightfield images. Using CellPose-SAM to segment single cells in conjunction with CellProfiler^43^ pipeline we evaluated how deep learning self-supervised models trained on image tiles compare with traditional phenotypic profiling based on hand-engineered features extraction applied to single cells. In this baseline assessment, we used CellPose-SAM to segment single cells and CellProfiler pipeline to extract features, which then underwent our best performing normalization method (MAD + Harmony/FOV), mAP and mAP-ES calculation. In the intermediate doses, CellProfiler features outperformed the cell count baseline for activity; however, CellProfiler baseline on live cells in brightfield overall provided results on par (in latest three timepoints) or lower (on first two timepoints) than DINO model without special adjustments in loss function, and substantially lower than the best performing PA + DINO + Barlow + XB model (see Fig. 2A,B). Overall CellProfiler baseline is less sensitive to phenotypic activity, as can be seen in Fig. SM2. These results can be partially attributed to the low signal-to-background ratio in live brightfield images; this is also in accordance with recent work showing superior performance of learned features from self-supervised learning compared to handcrafted features in Cell Painting datasets^26^.

### LCD outperforms baselines in MoA classification using multi-dose/multi-timepoint

**Fig. 3:**
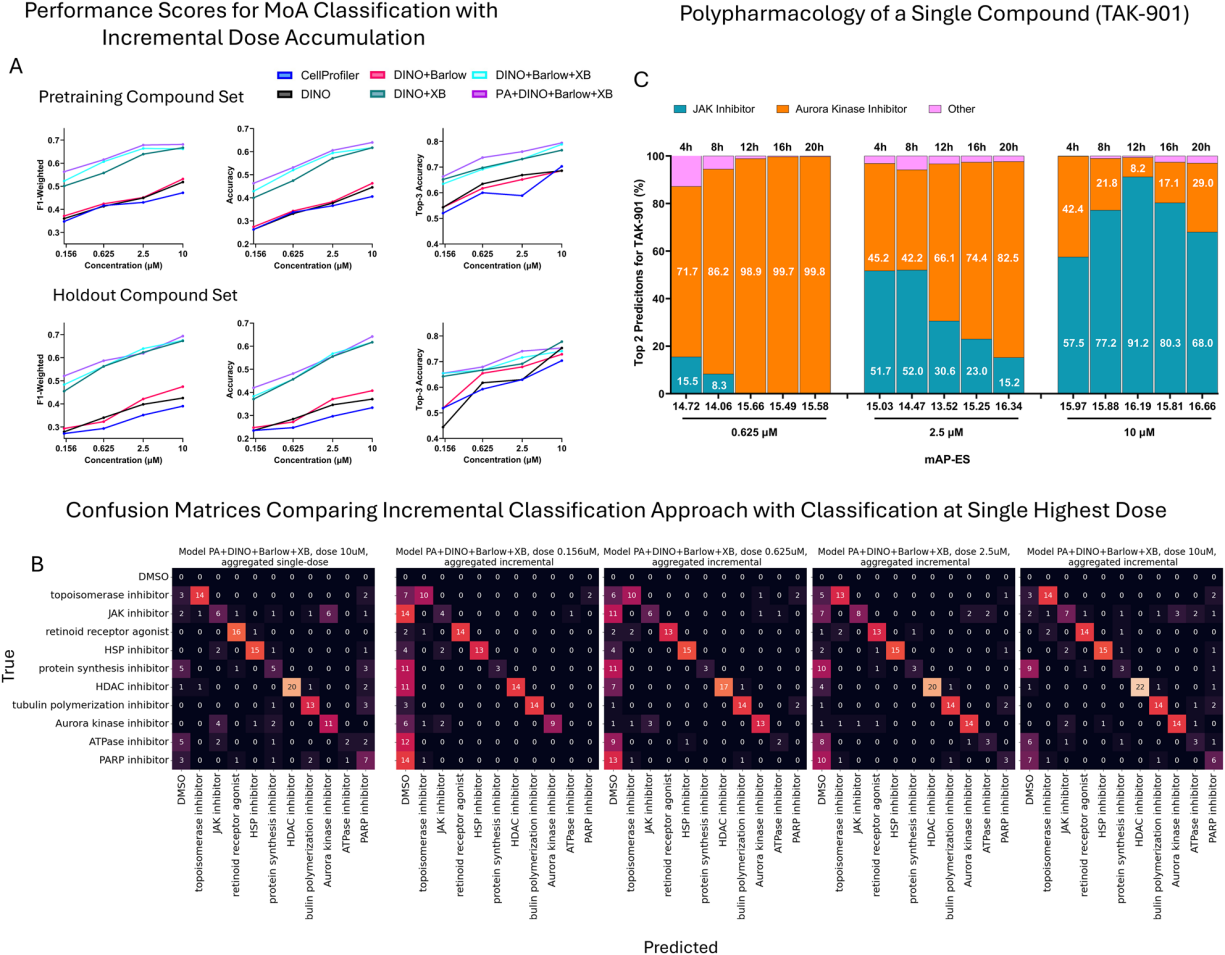
(**A**) F1-weighted, Accuracy and Top-3 Accuracy when doses (with all corresponding timepoints) were incrementally included into the MoA classification, on pretraining set (**A**, top row) and holdout set (**A**, bottom row). Aggregated linear probe provides best results when trained on active compounds identified by cross-batch-trained models, with plane-agnostic model performing best overall. (**B**) Confusion Matrices for PA + DINO + Barlow + XB model for single 10 μM dose, and for a series of incrementally aggregated doses. As more doses are included, less compounds are being identified as DMSO and confusions that arise on the later dose and timepoint get disentangled (e.g. Aurora kinase and JAK inhibitors). (**C**) Top-2 MoA predictions for compound TAK-901 across 3 highest doses and timepoints with predictions for all other MoAs are shown in pink. Across three top doses Aurora kinase and JAK inhibitors rank in the top-2 predicted MoAs across all timepoints, indicating that our phenotypic features allow to capture polypharmacology.

On the evaluation set, the multi-dose/multi-timepoint MoA classifier using PA + DINO + Barlow + XB outperformed other models including CellProfiler baseline, across nearly all doses and timepoints (Fig. 3A). (Downstream evaluation of MoA on the pretraining set was performed on 175 compounds which were recovered as phenotypically active by PA + DINO + Barlow + XB model.) To compare model performance, we used the chi-squared test for contingency tables, also known as McNemar’s test^44^. As expected, the primary improvement stems from cross-batch training (Table 2 and Table SM5), with our best model showing significant differences with baselines without cross-batch sampling. Once cross-batch sampling is added in the baselines, the difference is less significant; however, the plane-agnostic model consistently shows a trend toward higher accuracy across all doses (Fig. 3A, Table SM5). This is especially important at lower doses where phenotypes are visually similar to untreated controls and different from higher doses and late time points.

**Table 2:** F1-weighted and average accuracy scores of each MoA on the pre-training and holdout compound sets for different self-supervised models. On the pretraining set, downstream evaluation was performed on 175 compounds recovered as phenotypically active at 10 μM 20h by best performing model (PA + DINO + Barlow + XB).

		Pre-training compound set (compounds active at latest dose and timepoint)						Holdout compound set				
MoA	# compounds (total = 175)	DINO	DINO + Barlow	DINO + XB	DINO + Barlow + XB	PA + DINO + Barlow + XB	# compounds (Total = 81)	DINO	DINO + Barlow	DINO + XB	DINO + Barlow + XB	PA + DINO + Barlow + XB
Topoisomerase inhibitor	19	0.69	0.69	**0.76**	0.76	**0.76**	9	0.75	0.75	0.74	**0.78**	**0.78**
JAK inhibitor	19	0.30	0.29	**0.45**	0.43	**0.45**	10	0.29	0.47	0.56	0.59	**0.67**
Retinoid receptor agonist	17	0.19	0.27	**0.88**	0.82	0.85	9	0.33	0.50	**0.88**	**0.88**	**0.88**
HSP inhibitor	19	**0.88**	**0.88**	**0.88**	0.86	0.86	9	**0.71**	**0.71**	**0.71**	**0.71**	**0.71**
Protein synthesis inhibitor	14	**0.32**	**0.32**	0.30	0.30	0.29	6	0.40	0.20	**0.50**	0.33	**0.50**
HDAC inhibitor	25	0.77	0.77	**0.94**	**0.94**	**0.94**	7	0.44	0.44	**0.60**	**0.60**	**0.60**
Tubulin polymerization inhibitor	17	0.76	0.78	**0.82**	**0.82**	**0.82**	9	**0.84**	**0.84**	**0.84**	**0.84**	**0.84**
Aurora kinase inhibitor	18	0.53	0.56	0.74	0.74	**0.80**	7	0.33	0.38	**0.67**	**0.63**	**0.63**
ATPase inhibitor	12	0.25	0.27	0.25	**0.35**	0.32	7	0.00	0.22	0.46	**0.57**	0.53
PARP inhibitor	15	0.18	0.20	0.29	0.26	**0.40**	8	0.00	0.00	**0.67**	**0.67**	**0.67**
F1-weighted		0.52	0.53	0.67	0.66	**0.68**		0.43	0.47	0.67	0.67	**0.69**
Accuracy		0.45	0.46	0.62	0.62	**0.64**		0.37	0.41	0.62	0.62	**0.64**

.

We further explored the capability of our model to characterize the MoA landscape leveraging multi-dose and multi-timepoint information. In our classification we incrementally added information from each dose and provided a result as a weighted average of predictions per each dose and timepoint. Figure 3A shows that the overall classification (F1 and accuracy scores) performance increases with incremental accumulation of doses, and remains strong on holdout compounds unseen in both pretraining and downstream training. It is essential to recognize that predictions from single-dose and from incrementally accumulated doses provide complementary information about phenotypic patterns. In Fig. 3B we provide comparison of MoA classifier confusion matrices with only a single highest dose, and with per-dose predictions accumulated incrementally. For example, when compound potency is low at a low doses and phenotype is not sufficiently distinctive, our incremental approach tends to predict compound’s MoA as DMSO. If the same compound is taken at a single highest dose, the true underlying MoA is better recovered. These are the cases for protein synthesis, ATPase, and PARP inhibitors, with median mAP-ES across doses and timepoints 0.76, 0.38, and 0.53 respectively (see Table SM6). Our additional analysis showed that for all the compounds which were not predicted correctly by incremental approach for the highest dose, but predicted correctly in a single high-dose approach, correct MoA in incremental approach was still in top-3 predictions.

As an example of the advantages of incremental approach, Aurora and JAK inhibitors, while being largely confused in the latest dose, can be better distinguished from each other only by incorporating the entire time and dose landscape. Figure 3C shows the model capturing fine-grained MoA landscape of a single compound, with predictions as Aurora Kinase inhibitor and JAK inhibitor across three high doses and timepoints. This is consistent with previous studies showing poly-pharmacology of this compound (Aurora Kinase and JAK inhibitor^45^); importantly, our model reveals this critical insight based on live brightfield imaging only without the need for more sophisticated assays.

### Emergence of cellular semantics in attention mechanism

**Fig. 4:**
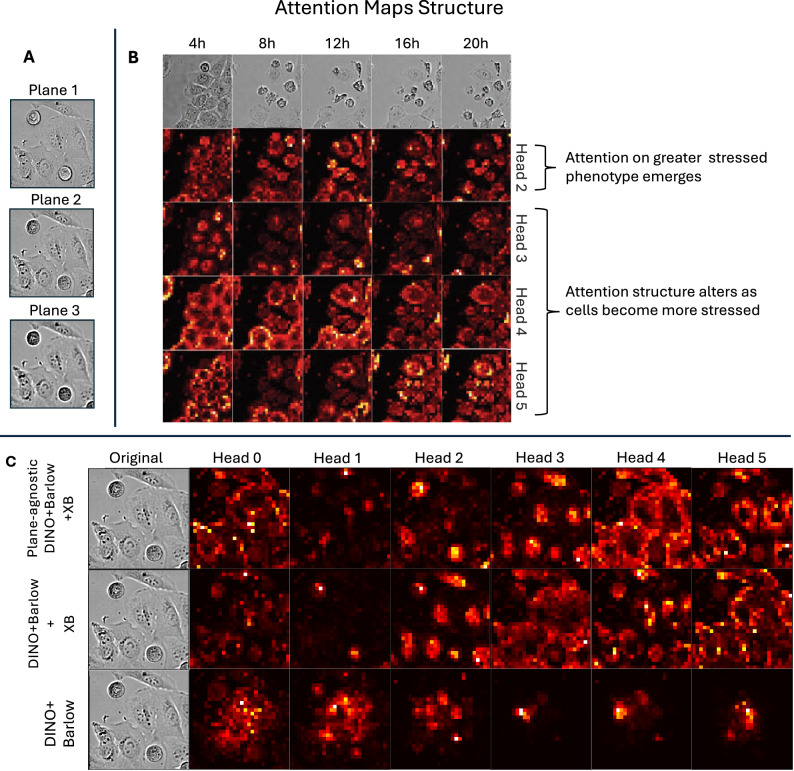
(**A**) Three brightfield planes from the same image tile exhibiting variation of focus between the planes. (**B**) Visualization of four exemplar attention heads from the last layer of Plane-agnostic DINO + Barlow + XB model for a single tile captured across multiple timepoints. Progressive destruction of cell structure is reflected in the reorganization of the model attention structure. As attention structure in live cell specific heads (3,4,5) gets altered, focus of head 2 on greater stressed cells emerges. (**C**) Visualization of attention mechanism from the last layer of ViT/s trained with three exemplar self-supervised approaches. Model focuses on cell-specific features of the image, such as cell nuclei (top row: head 3, middle row: head 2), cell area (top row: head 4, middle row: head 3), and cytoplasm (top and middle row: head 5). Attention maps matching subcellular structures are more distinctively defined in plane-agnostic-trained model. Cell-specific features do not emerge when trained without cross-batch loss (bottom row), potentially due to overt focus on technical variations.

Attention maps in Fig. 4 show that cross-batch training enables the model to distinguish meaningful cellular regions from the background, focusing on features like cell nuclei, cell area and cytoplasm, a capability previously demonstrated in self-supervised models for general vision^18,19,25,46^. In Fig. 4B, as cells degrade under treatment, attention maps reflect structural changes, revealing how the model’s focus reorganizes in response. Qualitatively, as demonstrated in Fig. 4C, the plane-agnostic ViT highlights these components more distinctly.

### Self-supervised vision transformer enables unsupervised live cell nuclei detection and counting

**Fig. 5:**
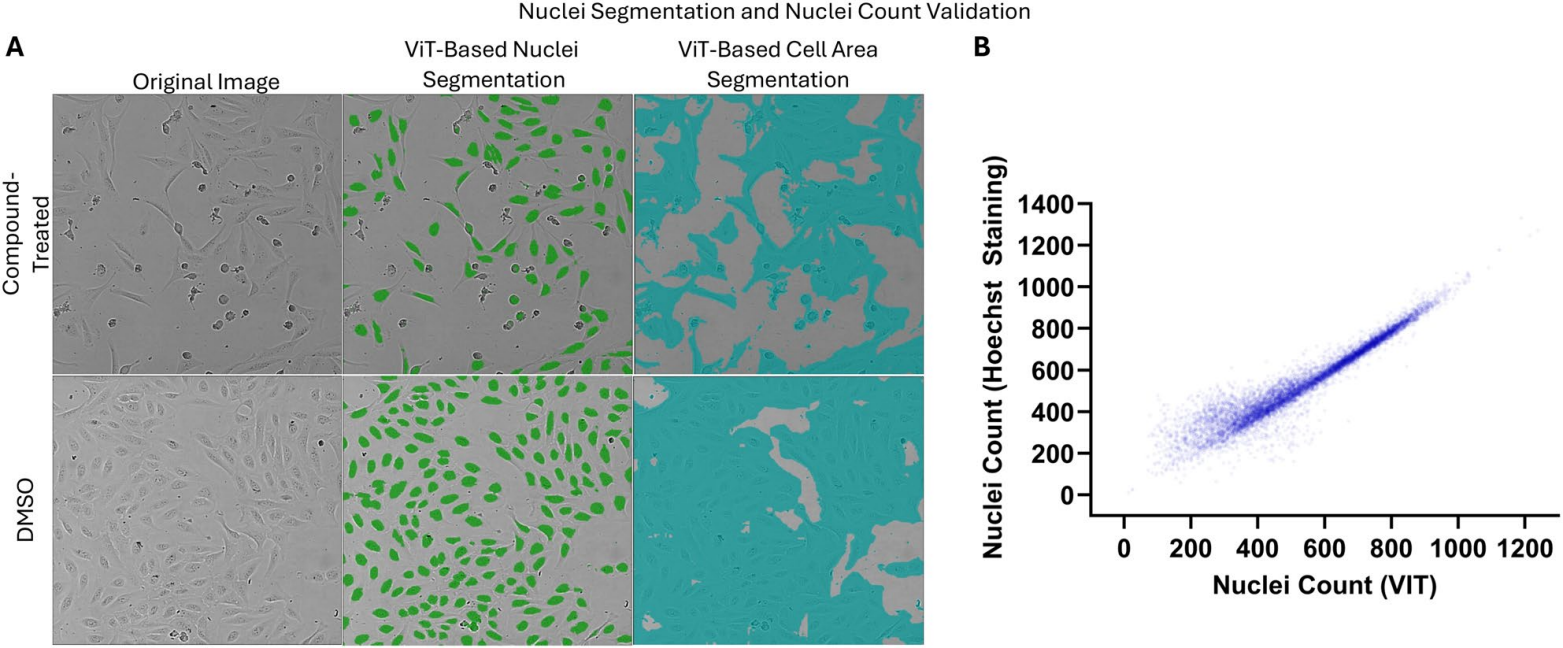
(**A**) Examples of compound-treated, and DMSO image, together with the results of unsupervised live nuclei (green prediction mask) and cell area segmentation (blue prediction mask) based on ViT patch-level features. (**B**) Plot of nuclei count per well based on ViT segmentation vs. Hoechst staining. The plot shows high agreement between nuclei count from the two modalities, with a Pearson correlation coefficient of 0.957.

Unsupervised object segmentation based on the pre-trained ViT patch-level features, and trained on the same pre-training dataset allowed for delineation of live nuclei and cell areas in the evaluation dataset (Fig. 5A). The algorithm was validated against Hoechst staining and segmentation of stained channel using commercially available Signal Image Artist software (SImA, Revvity, see Supplemental Materials). In Fig. 5B, our results show nuclei count by SImA and our nuclei detection method were highly correlated (Pearson’s r = 0.957). In addition, when measuring the segmentation mask overlap using intersection-over-union (IoU) metric, our results show substantial agreement with a median value of 0.566 (see Supplemental Materials, Fig. SM1 and section “Validation of self-supervised, label-free nuclei detection”). See Methods section for implementation details.

Besides live nuclei count, our pipeline allows for the confluency segmentation. Given confluency and nuclei count are considered two coarse metrics for cell death assays, we computed Inhibition Effect for each compound at a well level as $\% (Inhibition) = 100 * \frac{{mean(Metric_{DMSO} ) - Metric_{compound} }}{{mean(Metric_{DMSO} )}}$, where Metric is either Nuclei Count or Confluency. We found that Inhibition Effects calculated based on confluency and live nuclei count were highly correlated, showing Pearson’s r = 0.83, p < 0.001.

### Additional examples of downstream tasks enabled by LCD

In the section “Temporal imaging of cell recovery after drug withdrawal” of Supplemental Materials, we present an additional example of how LCD pipeline can be used to enable tracking cell recovery trajectories after drug withdrawal using phenotypic profiling. It demonstrates the ability of the model to capture meaningful time-resolved trajectories of phenotypes (Figs. SM4 and SM5).

## Discussion

We present for the first time a multi-dose and -timepoint end-to-end pipeline for mapping cell dynamics and recognizing live cell states, based on a novel self-supervised model trained on live brightfield imaging dataset. Our novel pipeline of Live Cell Dynamics (LCD) enables phenotypic profiling based on label-free imaging. Our approach recognizes overt and subtle—not visually obvious—phenotypes of complex live cell states, demonstrating superior sensitivity to detect compound activity. Furthermore, it captures MoA signals with generalizability to a holdout dataset of compounds unseen by the model, offering a novel avenue for scalable, cost-effective drug discovery based on dynamic phenotypic profiling of live cells.

### Self-supervised training: plane-agnostic cross-batch augmentation learns fine-grained features, cellular semantics and enables attention-based nuclei detection

We show that cross-batch DINO training reveals meaningful cellular regions in attention maps, with plane-agnostic training sharpening them further (Fig. 4). This likely reflects improved modeling of focal variation across depths—unlike Gaussian blur, which uniformly approximates out-of-focus effects. We further demonstrate, that simply removing blur in the augmentation leads to suboptimal results (see Supplemental Materials, Table SM2). As a result, the model better captures subtle phenotypic signals and can generalize more effectively to datasets with greater depth resolution or varying numbers of brightfield planes.

Compared to conventional feature extractors like CellProfiler, DINO features are less interpretable. To assist with feature interpretation, virtual staining has been developed as an alternative approach to bridge label-free inputs with biologically meaningful signals, even though their generalizability remains to be further validated^35,47,48^. Here we show our fine-grained features extracted from images enable accurate unsupervised nuclei counting—requiring no manual nor any florescent-based annotations—that captures biological relevant structures directly from raw brightfield data. We experimentally validate the performance of nuclei counting using stains, showing that the LCD vision transformer captures cellular semantics with high biological relevance. Validated nuclei detection algorithm allows for a stain-free, continuous nuclei count measurement in cell viability assays solely from live brightfield images. This is accomplished without costly manual annotation or additional data labeled collection.

### Replicate-based, batch effect-aware normalization and mAP metric improve Live Brightfield phenotypic activity detection

We proposed mAP-ES, a novel metric that captures mAP distributions to detect cell corresponding to compound activity relative to the experimental null distribution of negative controls. It accounts for real batch effects captured by negative controls and allows to comprehensively characterize activity across dosages and timepoints for each compound (Fig. 2B, Fig. SM2). mAP-ES is inherently designed for robust characterization of multiple treatment replicates, and is predominantly enabled by rapid, cost-effective acquisition of live brightfield data.

### Multi-dose and -timepoint MoA classification recovers Poly-pharmacology

Our MoA results indicate that predictions from a single dose and an incrementally accumulated set of doses can provide complementary views on compound phenotypic patterns (Fig. 3B). While for some compounds single-dose prediction may be beneficial (e.g. low-potency compounds are better predicted at a high dose), relying only on highest doses can confuse MoAs with similar late-stage effects (e.g. Aurora kinase and JAK inhibitors, Fig. 3B). Including early doses and timepoints incrementally into classification reduces this confusion by emphasizing early phenotypes; we note that here we implemented a simple approach to incrementally add doses, even though more sophisticated classifiers to selectively leverage dose- and time-series can be developed in the future.

Polypharmacology—dose- and time-dependent mechanisms of multiple biological targets—is a well-established yet difficult-to-unravel attribute of many small molecules^49^. For example, TAK-901, originally advanced as an Aurora-Kinase inhibitor, has been subsequently shown, following extensive kinome surveys and pathway-specific reporter assays, to potently inhibit JAK3 and attenuate JAK/STAT signaling^45,50,51^. Our Live Cell Dynamics brightfield pipeline recovers this poly-pharmacology through precise mapping of subtle cell state changes in a single, low-cost, reagent-free imaging experiment, detecting both phenotypic signals of Aurora and JAK-pathway inhibitions, and their evolution across three highest doses and time points (Fig. 3C). Thus, for TAK-901, Live Cell Dynamics allows poly-pharmacologic discoveries to be concluded within hours of live imaging and automated analysis, which would otherwise require extensive bench work and intricate biological assays. This highlights the power of live-cell time-continuous brightfield phenotypic profiling as a rapid and cost-effective first-line strategy for uncovering subtle, multi-pharmacological mechanisms often missed in snapshot-based assays.

Previous research demonstrated improvement in MoA classification by incorporating a series of timepoints at a single dose^36^. In our work, we demonstrate improvement in MoA classification by incorporating both dose and timepoint series, and its utility for classification across ten MoAs, compared to six in previous work. Additionally, in contrast with previous work, we demonstrate that our model successfully tackles live brightfield modality challenges in pre-training stage, not only enabling phenotypic activity or MoA classification, but also downstream unsupervised nuclei detection.

### Limitations

This proof-of-concept study uses only a single cell line and a limited collection of compounds for pre-training and evaluation. In our current work, we showed the model performance is transferrable in a holdout test set of 81 compounds; to further assess its generalizability, an expansion of both cell types and number of compounds will be explored in the future. Expansion to new cell types and compounds may require revisiting the hyperparameters, e.g. tile crop size, DINO and Barlow head layer dimensions, and dataset curation. Future work to further explore its capability for biological discoveries, including polypharmacology, is also warranted.

## Conclusion

In this work, we built Live Cell Dynamics—an end-to-end live brightfield pipeline for phenotypic feature extraction, live nuclei detection, and prediction of activity, MoA and polypharmacology. We demonstrated that a tailored self-supervised method trained and applied to a large multi-dose and -timepoint live brightfield dataset enables recovery of early cell state phenotypes, and bioactivity disentangled from cell viability and cytotoxicity. To our knowledge, we also introduced a first live brightfield MoA classifier based on cell state changes across multiple doses and timepoints, and demonstrated LCD’s capability to reveal compounds’ poly-pharmacology. This represents a significant advancement in high-throughput live cell state-based drug discovery. Methods to train the foundation model and assess robustness against technical effects—cross-batch sampling, normalization, and mAP-ES metric—broadly applicable to other modalities such as fluorescence imaging. Beyond this application, this approach can be extended to other areas of biology with evolving dynamical ecosystems, including target identification, disease diagnosis and staging, elucidation of disease mechanisms, modulation of cell phenotypes for drug discovery, assessment of cell state spaces for drugability and toxicity, and development of targeted therapies aimed at modifying or eliminating disease-associated cell states.

## Methods

### Dataset

For pre-training, we selected 189 compounds from ten MoA classes (ATPase, Aurora kinase, HDAC, JAK, HSP, PARP, protein synthesis, topoisomerase, tubulin polymerization inhibitors, and retinoid receptor agonists, see distribution in Table SM1) at 10uM similar to Harrison et al.^52^ and randomly distributed them in eight 384-well plates seeded with human osteosarcoma U2OS cells, with each plate containing all compounds, with median of 2 wells per compound (technical replicates) per plate. Using an Opera Phenix microscope (20× air, 2160 × 2160 pixels) images were captured at five timepoints (4, 8, 12, 16, and 20 h post-administration) with four fields of view (FOV) and three z planes (3.6u step size) per field for each well. We define a **replica** as one complete experimental run (seeding, administering compounds, and imaging all plates at all timepoints), while a **replicate** is each well receiving the same treatment. Seven replicas of the pre-training set were collected to account for biological replicate variability, totaling 430′080 raw images. After preprocessing, the pre-training dataset size was 5.7 M input images of 224 × 224 × 3 pixels(See Supplemental Materials for details). For multi-dose evaluation, a single replica at each of three doses (0.156, 0.625, 2.5μM) and two replicas of 10μM doses were acquired using same protocol, totaling 307,200 raw images. For holdout set, 89 additional compounds belonging to the same classes were distributed across four 384-well plates, and data from 81 of them passed quality control (122,880 raw images). Supplemental Materials contain details on cell preparation, data collection, data preprocessing and data amount.

### Self-supervised model training

We chose DINO^18^ with a ViT-small backbone^53^ as our primary algorithm based on three key advantages: its effectiveness in embedding space clustering^18^, capabilities in zero-shot learning, and successful applications in fluorescent cell imaging^23–25,38^. Our implementation builds on recent advances in addressing microscopy-specific challenges, incorporating the cross-batch loss and sampling strategy proposed by Haslum et al.^38^ to mitigate batch effects present in high-throughput fluorescent microscopy data, and the Barlow Twins loss function^22,38^, to combine sample-contrastive and feature-contrastive self-supervised methods^54^, for enhanced feature learning robustness.

To assess each loss component’s contribution, we conducted an ablation study across four model variants each trained for 60 epochs with a standard DINO training schedule^18^ with the start learning rate of 0.001 on the same pre-training dataset:

- Standard DINO loss (DINO)
- DINO + Barlow loss (DINO + Barlow)
- DINO + Cross-Batch loss (DINO + XB)
- DINO + Barlow + Cross-Batch loss (DINO + Barlow + XB)

### CellProfiler baseline

Additionally we performed an experiment to create a baseline from CellProfiler^43^ features extracted on a single-cell level, where single cell segmentation was extracted using the most recent CellPose-SAM^14^ model. We share the Cell Profiler pipeline with the parameters we used in the code repository accompanying the paper. On a single-cell level, we extracted 284 features, which then were median-aggregated per image. The following steps (normalization, phenotypic activity and MoA classification) were identical for all self-supervised models and CellProfiler baseline.

### Plane-agnostic vision transformer as a data augmentation strategy

While standard DINO^18^ uses Gaussian blur for feature stability, we observed that brightfield z-stacks naturally offer varying focal sharpness. In this work, we used three z-planes (− 3.6, 0 and + 3.6 μm) covering the depth of field range for training, and a similar approach can be employed in other objective configurations. We perform an ablation study, to confirm that focal variability, not blur removal alone drives performance change (see Supplemental Materials, Table SM2).

We evaluate the augmentation strategy using our best performing loss function configuration:

 Plane-agnostic DINO + Barlow + Cross-Batch loss

 (PA + DINO + Barlow + XB)

(See Fig. 1 for the model architecture).

### Loss functions ratio and Barlow Twins decorrelation hyperparameter

When training with Barlow twins loss we experimentally found that strong decorrelation helps training, and the coefficient for off-diagonal Barlow Twins loss elements was set to 0.5; In the total loss function, DINO: Barlow Twins losses ratio was 1:1. For cross-batch training, two global crops were sampled from two different experimental replicas of the same compound at the same dose and timepoint. For each image serving as source for global crop, three local crops were sampled. In case of plane-agnostic training, plane was randomly selected for each crop.

### Downstream nuclei detection

Recent body of work^55,56^ demonstrated that features learned by self-supervised model can be informative for a completely unsupervised object discovery task. The work by Simeoni et al.^57^ demonstrated the possibility of segmentation and detection of salient objects from background, with a relatively simple unsupervised algorithm guided by the pre-trained attention maps and features, and a computer vision segmentation smoothing algorithm (Bilateral Solver^58^). We tested whether this algorithm could perform completely unsupervised live nuclei detection from brightfield images using our best-performing self-supervised training model patch-level features, and validated the algorithm against Hoechst staining (see Results section for training and validation results, and Supplemental Materials for details of biological experiment).

### Self-supervised model evaluation

Self-supervised learning can provide a universal feature extractor to determine cell state for diverse tasks. We validated this in live cell, brightfield microscopy, using a five-stage assessment pipeline at each combination of dose and timepoint:

1. Feature Extraction: Generation of embeddings from evaluation and holdout set at each timepoint and dose.
2. Normalization: Application of embedding normalization techniques.
3. Phenotypic Activity Assessment: Evaluation of feature space properties using Mean Average Precision (mAP^39^ and mAP-ES) metric, to estimate activity of each compound.
4. Benchmarking Phenotypic Activity against Nuclei Count as a Conventional Assay: segmenting and counting nuclei on each image, and comparing activity detected by nuclei count with activity based on mAP and mAP-ES metric.
5. Downstream Task Validation: Assessment of learning representations through MoA classification using linear probes over extracted features.

MoA classification per each dose and timepoint was then incrementally aggregated for MoA prediction (see “MoA classification” section).

### Embeddings normalization approaches

While batch effect normalization is well-studied in fluorescent imaging^59–61^, its effectiveness for live brightfield embeddings remains unexplored. Based on Arevalo et al.’s^59^ analysis, we selected the top-performing methods from three distinct families under comparable settings (single microscope, comparable amount of compounds, multiple replicas):

- fastMNN^62^, a nearest-neighbor-based method
- Harmony^63^, a mixture-model-based method
- Baseline, Arevalo et al. baseline method (using Median Absolute Deviation per plate normalization)^59^.

Additionally, a novel approach (“MAD+Harmony/FOV”) was proposed and evaluated as described below.

### *A novel batch correction approach: MAD* + *Harmony/FOV*

After features had been extracted, they were concatenated in the pandas dataframe with all the features per tile and corresponding metadata. As a first step, each measurement (plate imaged at a particular timepoint) was normalized separately to its DMSO wells using MAD + Robustize method, then all normalized embeddings are concatenated, and normalized using Harmony with groupings only at the level of experimental replica, and field of view. Pseudo code for the algorithm is provided in Algorithm SM1.

### Evaluation of phenotypic activity

To quantitatively assess the quality of the embeddings across different models and normalization techniques, we employed Mean Average Precision (mAP), a common measure of compounds’ phenotypic activity^39^. In our novel approach enabled by multiple replicates of the compounds, mAP for each compound is treated as an experimental distribution, rather than a single value, and a null distribution is constructed based on DMSO samples. Phenotypic activity is then defined as the strength of difference between mAP per compound and null distribution measured by Cohen’s d effect size^40^ (mAP-ES), with compound defined as active if mAP-ES ≥ 0.8 (corresponding to large effect size in statistical literature^40^).

### Mean average precision effect size (mAP-ES)

mAP^39^ quantifies how distinctly a compound’s phenotypic response can be distinguished from negative controls in the embedding space. To calculate it, for each query well with perturbation, its positive and negative pairs should be defined^39^. For compound treatments we defined its positive counterparts as three wells with the same treatment, but taken from a different randomization plate from a different experimental replica (presumably strongest batch effect difference). Negative pairs were defined as 31 randomly sampled DMSO wells from the same plate as the query well (presumably smallest batch effect difference).

In the same vein, the null distribution of mAP for DMSO wells was generated: a query DMSO well was randomly selected, and three DMSO wells from different plates and experimental replicas were defined as positive pairs. Thirty-one DMSO wells from the same plate were selected as negative pairs. By randomly sampling query well, positive, and corresponding negative pairs 1000 times, the distribution of mAP for each compound, and null distribution of mAP for negative controls was created.

**Fig. 6:**
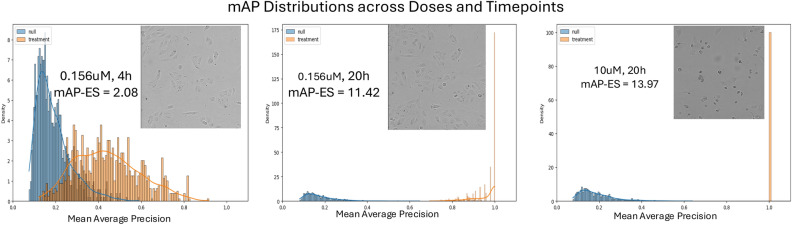
Example of mAP distributions for null and treatment (compound TAK-901) distributions at three doses and timepoints for different activity levels.

Phenotypic activity was defined as the strength of difference between mAP per compound and mAP null distributions measured by Cohen’s d effect size^40^ (mAP-ES). This approach treats mAP per compound as a random value, and null distribution of mAP for DMSO wells by design accounts for batch effects present in the dataset. Example of mAP distributions are shown in Fig. 6.

### Benchmarking phenotypic activity against nuclei count

Cell count is a recently proposed baseline for assessing the added value of phenotypic profiling, as many activity benchmarks correlate strongly to viability and can be predicted from a single cell count feature^41^. Live cell count per field of view was extracted for each image using a nuclei detection algorithm. Normalized cell count was computed as Z-transform of the cell count based on statistics for DMSO wells for each plate at each timepoint, and for each perturbation, the 95% confidence interval for the median of all replicates (without any prior aggregation) was calculated using bootstrap method. Compound was deemed active if upper bound of the confidence interval was less than zero (implying that for DMSO well, normalized cell count is equal to zero). Number of compounds deemed active based on the cell count was then compared to number of active compounds based on mAP-ES.

### MoA classification

We further evaluated the representations quality via MoA classification using linear probes^33^, a standard approach for self-supervised learning that tests whether embeddings contain linearly separable class information. Five-fold cross-validation was employed for the pretraing compound set, and for holdout set, the classifier was trained on active compounds from pretraining set.

To address potential polypharmacology^49^ and varying dose responses, we trained a separate MoA classifier on compounds active per each dose-timepoint condition and then generated final MoA predictions by taking a weighted average of their softmax outputs. Dose contributions were evaluated by incrementally adding doses, with classifiers trained on activity-filtered data per condition.

## Supplementary Information

Supplementary Information.

## Data Availability

Four biological replicas of holdout dataset at different doses, embeddings for the evaluation dataset and model weights are available at AWS S3 bucket: s3://soley-lcd-scirep . The full evaluation set is available upon request. Embedding extraction, normalization, mAP-ES and linear probe code are available at the following link:https://doi.org/10.5281/zenodo.17957968. Code, data and model weights are for use under a research non-commercial use license.
