# Supplementary material for: A self-supervised machine learning pipeline for extracting information from live cell images at multiple doses and timepoints
Source: Sci Rep. 2026 Jan 7;16:891. doi: 10.1038/s41598-025-32685-5 (PMC12783685; doi:10.1038/s41598-025-32685-5)
Supplement: Supplementary file 1 — Supplementary Information. [file 41598_2025_32685_MOESM1_ESM.pdf]

Data collection and preprocessing

Cell preparation

Human osteosarcoma U2OS cells were obtained from ATCC and maintained in cell culture T225 flasks (Falcon 353138) at 37°C with 5% CO_2 and cultured in McCoy's 5A Medium, supplemented with 10% fetal bovine serum (FBS) and 1% penicillin-streptomycin . Cells were refed with fresh medium every 3-4 days until 80% confluency when they were either harvested for experiment or sub-cultured as new passage. Cells were trypsinized with 0.25% trypsin-EDTA, pooled and counted using an automated cell counter (Corning CytoSmart 6749). For experiments using plates with cyclic olefin polymer bottom, cells were seeded into 384-well assay plates (Revvity PhenoPlate 6057302) at a density of 1700 cells/well (50 uL at 34,000 cells/mL) using the EL406 microplate reagent dispenser (Agilent-BioTek); for experiments using plates with glass bottom, cells were seeded into 384-well assay plates (ibidi μ-Plate, 88407) at a density of 1900 cells/well (50 uL at 38,000 cells/mL) using the EL406 microplate reagent dispenser (Agilent-BioTek).

Compound preparation

Majority of compounds were sourced from MedChem Express as DMSO aliquots at 10 mM stock. Some molecules were purchased as solid powder and dissolved in DMSO to make 10 mM stock. Compound stocks were vortexed and sonicated to ensure complete dissolution into DMSO solvent. Stock compounds were cherrypicked and plated into 384-well polypropylene plates (Greiner 784261), using automated liquid handling system (Revvity JANUS G3 workstations). Compounds were also made into 2-fold dilution series covering 10 concentrations for dose response experiments. All compounds were stored at -40°C until use, with minimal freeze-thaw cycles.

Data collection

For the pre-training dataset, seven experimental replicates were collected. Each replica consisted of eight plates, imaged at five timepoints, resulting in 430’080 original images in total. A total of 189 compounds were distributed across eight plates, with each plate containing all the compounds. Then, at each of four doses (0.156uM, 0.625uM, 2.5uM and 10uM), one experimental replica was collected in the same way, constituting evaluation dataset (307’200 raw images in total). The same 189 compounds were selected for the analysis in the evaluation dataset. For the holdout set, originally, 89 compounds were distributed and imaged (122’880 raw images in total), and 81 compounds were retained after Quality Control procedure*.

Sixty-four DMSO wells were designated on each plate, including 32 wells on left and right side of the plate (the first and the last column), and 32 wells randomly distributed inside the plate. Assuming that outer DMSO wells are more susceptible to technical variation (e.g. edge effects of humidity and temperature), 32 outside DMSO wells were discarded from all steps of data processing, including pre-training, feature extraction, normalization, phenotypic activity estimation, and MoA classification.

Table SM1. Number of compounds per Mechanism of Action passed QC

Mechanism of Action	Pretrain # of compounds	UAT # of compounds
HDAC inhibitor	25	7
topoisomerase inhibitor	23	9
HSP inhibitor	19	9
JAK inhibitor	19	10
tubulin polymerization inhibitor	19	9
retinoid receptor agonist	17	9
PARP inhibitor	18	8
Aurora kinase inhibitor	18	7

* Quality Control (QC) procedure included visual review of the images at 10μM dose and 20h timepoint. Visual evidence of the crystallization of the compound, which can happen at high concentration, was the criterion for attrition, as well as visual inconsistency of compound activity (e.g. when for the same compound some wells exhibit strong activity, and some visually look inactive, which may be due to technical batch variation).

protein synthesis inhibitor	16	6
ATPase inhibitor	15	7

Data preprocessing

Each image was downsampled to 1120x1120 pixels using skimage library, and illumination-corrected via a standard procedure.[1] Images were then tiled into 5x5 grid (25 tiles per FOV), with tile size of 224x224, with autocontrast applied to each tile. Cell area segmentation procedure following Cepa et al.[2] was then applied to tiles, and fraction of cell area (confluence score) was computed per tile. To create a final dataset, attrition of tiles with confluence below 50% was performed. A cloud based automated preprocessing pipeline including all these steps was created and enabled conversion of raw microscope 16-bit TIFF images into zarr dataset of image tiles which was used for training and inference, totaling 5.7M tile images for pre-training, 3.2M for evaluation, and 1.1M for holdout. Same pipeline was used for both pre-training, evaluation and holdout dataset. All self-supervised models presented in this work were trained on the pre-training dataset.

Compound list

Compounds were split into 5 folds using scikit-learn StratifiedKFold method such that balance of number of compounds per MoA was kept approximately the same across folds, and each compound would belong to one and only one-fold. MoA classification was then done in 5-fold cross-validation manner, where each fold iteratively became the test split, and four other folds constituted training split.

For the list of active compounds, same procedure of splitting into five folds was repeated separately, with the goal of keeping MoA balance across folds for active compounds too.

Ablation study for plane-agnostic training

A potential alternative explanation for the improvements achieved by plane-agnostic ViT model is that Gaussian blur at the augmentation stage is suboptimal, and the model simply benefits from the absence of blurring operation. We examined such explanation by

training a separate model with DINO+Barlow+XB loss function taking as an input a 3-plane image, but without any blur in the pipeline. Performance on phenotypic activity evaluation is shown in Table SM2. Performance of the model without blur does not improve above its baseline and stays far from exceeding the plane-agnostic model variant.

Table SM2. Performance of the self-supervised model without Gaussian blur compared to the model with blur and the plane-agnostic version under best performing (MAD+Harmony/FOV) normalization method.

**Model, Training Loss, Augmentation**	**mAP mean**
DINO	0.45
DINO+Barlow	0.46
DINO+XB	0.61
DINO+Barlow+XB	0.64
PA+DINO+Barlow+XB	**0.69**
DINO+Barlow+XB – no blur	0.63

Normalization algorithm

Algorithm SM1 Algorithm for MAD+Harmony/FOV Processing

Require: *df* : Dataframe containing all measurement features per tile (*replica*, *timepoint*, *dose*, *plate*, *FOV*)

```
1. Per-plate normalization (MAD Robustize) w.r.t DMSO
for replica 1,2, ... do
    for timepoint 1,2, ... do
        for dose 1,2, ... do
            for plate 1,2, ... do
                for FOV 1,2, ... do
                    Define df_measurement based on replica, timepoint,
                    dose, plate, fov filters
                    MAD Robustize the df_measurement w.r.t. DMSO
                end for
            end for
        end for
    end for
end for
2. Harmony with only replica and field of view as metadata groups
  Store metadata
  Drop all metadata besides replica, fov
  Run Harmony with replica, fov groupings
  Concatenate metadata with Harmony-transformed embeddings
Result: MAD_Robustize, then Harmony-transformed embeddings on tile level
```

Validation of self-supervised, label-free nuclei detection

We validated the performance of ViT nuclei detection and counting by comparing nuclei count from ViT segmentation with that based on Hoechst staining. In this validation experiment, U2OS cells are first treated with compounds at 10 μM; for compound treatment, eight plates containing 190 compounds (see "Data Collection" section) were used, and we acquired 3 replicates of each plate set. To obtain nuclei count, live cells in the treated cell plates were stained with Hoechst staining, prior to live brightfield and fluorescence imaging at 20 hours. In live brightfield images, the ViT segmentation algorithm was applied, and the nuclei count was computed. In fluorescence images, stain-based nuclei count was calculated based on nuclei segmentation using the SImA software (Signals Image Artist, Revvity).

In addition, we also computed the intersection-over-union (IoU) values for each field between Hoechst staining segmentation of nuclei, and our ViT-based segmentation of nuclei. Our results show substantial agreement, with a median of 0.566 and an interquartile range of 0.469 – 0.595; an example image is also shown (Fig. SM1). Our results show that the emerging cellular semantics from the foundation model allows for accurate nuclei

detection, and we believe the segmentation performance can be further improved, potentially with finetuning and annotations.

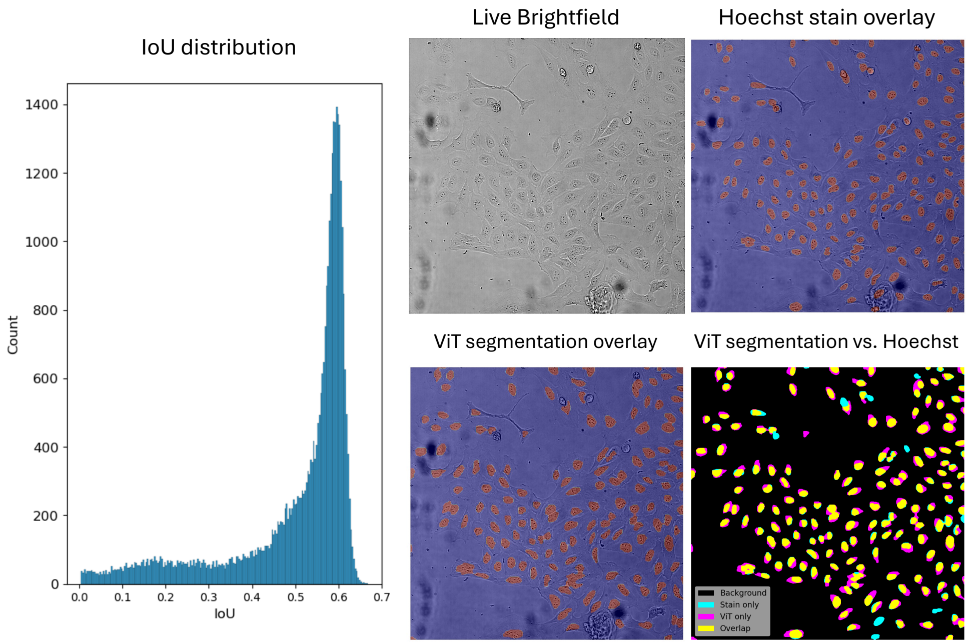

Figure SM1. Distribution of IoU values calculated between Hoechst staining segmentation of nuclei, and our ViT-based segmentation of nuclei. An example image together with the segmentation masks are shown on the right panel (top left: original live brightfield image; top right: Hoechst staining nuclei mask overlay on brightfield image; bottom left: ViT nuclei segmentation mask overlay on brightfield image; bottom right: ViT nuclei segmentation mask overlay on Hoechst staining nuclei segmentation).

Statistical Comparison of Model Performance

mAP performance comparison

We statistically compared mAP differences between models. For that we compared per-compound mean mAP for each model using Wilcoxon paired signed-rank test. P-values were then corrected with Bonferroni correction. mAPs between each pairs of models were found to be significantly different.

Table SM3. Bonferroni-corrected p-values between pairs of models.

	DINO	DINO+Barlow	DINO+XB	DINO+Barlow+XB	PA+DINO+Barlow+XB
DINO	-	0.0032**	6.78e-29**	1.78e-29**	3.1e-30**
DINO+Barlow	-	-	1.18e-28**	4.51e-29**	5.65e-30**
DINO+XB	-	-	-	1.66e-28**	1.71e-29**
DINO+Barlow+XB	-	-	-	-	2.42e-28**
PA+DINO+Barlow+XB	-	-	-	-	-
CellProfiler†	1.36e-18**	4.71e-20**	4.01e-30**	1.96e-30**	2.73e-31**

In the same vein, normalization methods were compared using Wilcoxon paired signed-rank test. The results are presented in Table SM4.

Table SM4. Bonferroni-corrected p-values between pairs of normalizations.

	MAD	fastMNN	MAD+Harmony	MAD+Harmony/FOV
MAD	-	1.77e-22**	4.6	6.75-31**
fastMNN	-	-	10.0	7.17e-30**
MAD+Harmony	-	-	-	8.10e-31**
MAD+Harmony/FOV	-	-	-	-

Classification performance comparison

We performed McNemar test[3] for comparison of accuracies between different pretraining approaches. The results are presented in Table SM5.

Table SM5. Comparison of classification performance between

PA+DINO+Barlow+XB and baselines

**Model**	**# Correctly predicted compounds (total=175)**				**McNemar test p-value between PA+DINO+Barlow+XB and the model**			
	**0.156μM**	**0.625μM**	**2.5μM**	**10μM**	**0.156μM**	**0.625μM**	**2.5μM**	**10μM**
CellProfiler	46	59	64	71	2.27e-08	1.52e-08	2.51e-10	1.06e-09
DINO	46	58	66	78	9.08e-09	2.28e-08	6.98e-10	1.52e-08
DINO+Barlow	48	60	67	81	2.54e-08	6.34e-08	1.17e-09	7.12e-08
DINO+XB	70	83	100	108	0.006	0.004	0.041	0.134
DINO+Barlow+XB	75	91	104	108	0.077	0.683	0.617	0.134
**PA+DINO+Barlow+XB**	**81**	**93**	**106**	**112**				

† Only a single normalization method ("MAD+Harmony/FOV") was used to compare CellProfiler with other baselines.

Distributional analysis of activity

Our proposed metric, mAP-ES is estimated for each compound at each dose and timepoints. In our work we looked at sensitivity at the threshold of 0.8 for mAP-ES, based on conventional interpretations of Cohen's D. In Fig. SM2 we show an extensive analysis to compare sensitivity of activity detection at different thresholds. Plane-agnostic model outperforms other baselines across the entire range of thresholds.‡

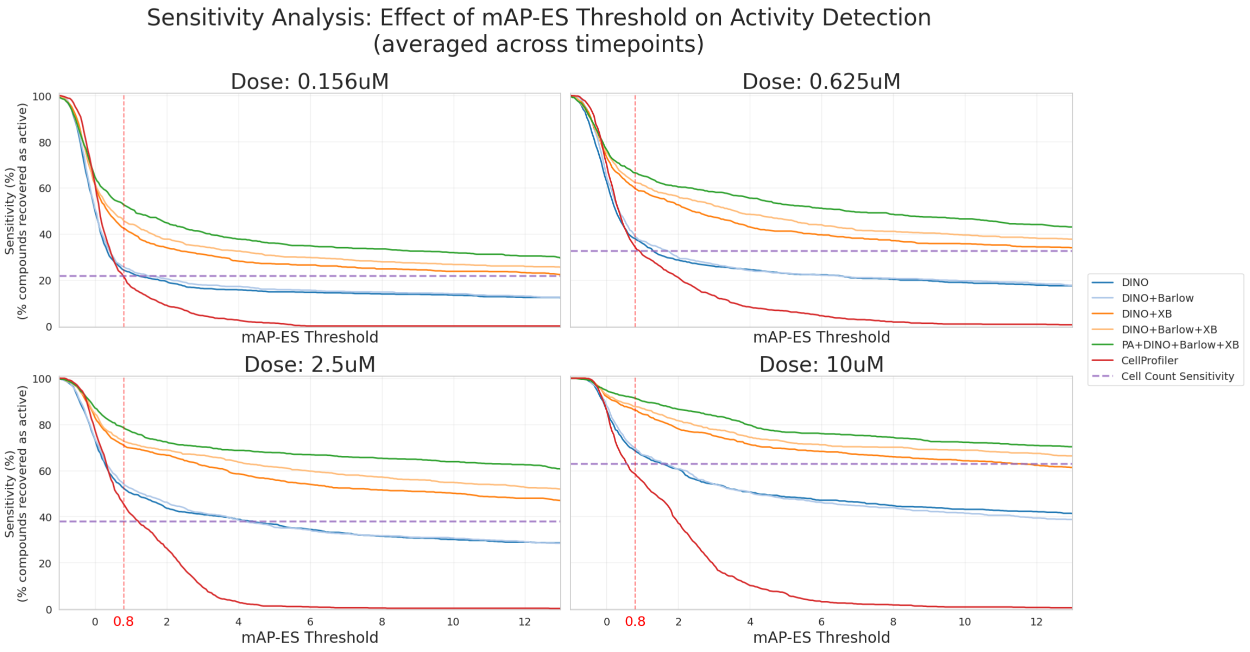

Fig. SM2. Sensitivity of phenotypic activity detection across mAP-ES thresholds.

Additionally, the nature of our approach to mAP allows for distribution-based definitions. A measure of activity as 'compound mAP 95% confidence interval not covering null mAP distribution mean' can be defined Our results show, that based on such definition, activity based on mAP distributions outperforms the Cell Count baselines, specifically on the lower doses and earlier timepoints (Fig. SM3)

‡ Due to the statistical nature of mAP-ES computation, for some compounds with very low potency (indistinguishable for DMSO) mAP-ES can be below 0.

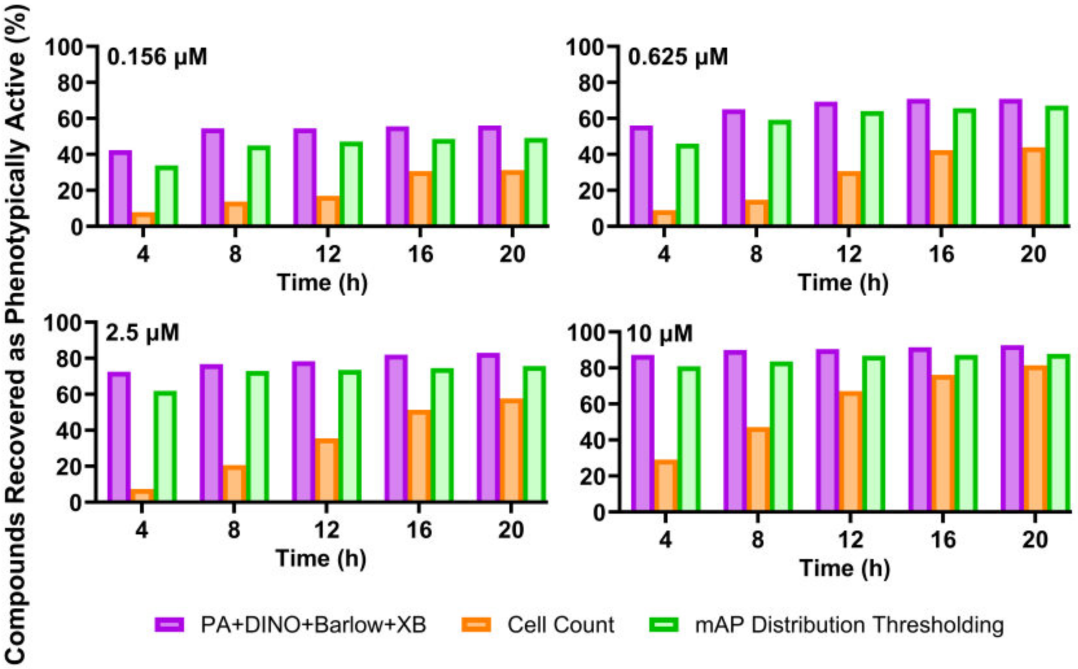

Fig. SM3. Comparison of three ways to approach compound activity: mAP-ES (Cohen's D) threshold of 0.8, Cell Count (both presented in the main paper), and compound mAP distributions 95% CI not overlapping with null mAP mean.

MoA potencies variability

Compounds within different MoAs in our dataset demonstrated different level of potencies. Specifically there were three MoAs (protein synthesis, PARP, ATPase inhbitors) where compounds exhibited overall low potency across the range of doses and times. In Table SM6 we provide median mAP and mAP-ES across doses and timepoints for all compounds within each MoA.

Table SM6. Median mAP and mAP-ES across all compounds, doses, and timepoints within MoA, demonstrating activity variability.

Mechanism of Action	Median mAP	Median mAP-ES
ATPase inhibitor	0.208734	0.383355
Aurora kinase inhibitor	1.000000	14.472145
HDAC inhibitor	1.000000	14.472145
HSP inhibitor	1.000000	15.025266

JAK inhibitor	0.971860	12.534840
PARP inhibitor	0.230691	0.535176
protein synthesis inhibitor	0.247464	0.755329
retinoid receptor agonist	0.933766	10.181095
topoisomerase inhibitor	1.000000	14.472145
tubulin polymerization inhibitor	1.000000	15.253040

Temporal imaging of cell recovery after drug withdrawal

Despite being trained on single images, in the inference mode, LCD feature extractor can be used to unravel temporal trajectories in the presentational embedding space. We tested the use of this hallmark of LCD model in a experiment of cell recovery after drug withdrawal, where three groups of live cells were treated with three different doses of Bortezomib (a protein degradation inhibitor as the stressor) respectively; after 4 hours, media containing the stressor was washed out, and then cells were imaged for 17 hours with 1 hour sampling interval. It is important to notice that neither Bortezomib nor any other compound from protein degradation inhibitors class were included in pre-training or holdout set. Our results demonstrate the ability of the model to differentiate trajectories of compound phenotype evolution between different doses, and reveal the samples that converged on the trajectory of non-treated cells after stressor removal in the cell recovery after drug withdrawal experiment.

Data collection

A single 384-well plate was used in the experiment. Cell preparation followed the same protocol as the pre-training and holdout sets. The plate was partitioned into two equal (left and right) parts of 12 columns each to test two different experimental conditions. In each part, three doses (0.625, 2.5 and 10uM) of Bortezomib were tested in 8 randomly positioned technical replicates. 32 wells were designated as untreated controls in each half of the plate. Columns 1-12 were washed gently using an AMX cassette on the Bio-TEK

Multiflo, four hours post treatment. Columns 13-24 were not washed. After the wash step, plates were incubated at 37oC and imaged every 4 hours for 72h, resulting in 17 readouts of entire plate. Imaging protocol was the same as for pretraining and holdout sets.

Feature extraction

Images underwent the same preprocessing steps as the pretraining and holdout sets (see "Data Preprocessing" section above), and then fed into LCD backbone feature extractor of the best-performing model (PA+DINO+Barlow+XB).

Visualization and Statistical Analysis

After embeddings were extracted, two-dimensional UMAP was performed separately on the embeddings from unwashed and washed parts of the plate. Visualizations of the UMAP plots, color coded by timepoint are shown in Fig. SM4. It demonstrates the ability of the LCD model to produce time-resolved trajectories of cell phenotype dynamics. While trajectories of untreated cells and treated but not washed cells strongly diverge (Fig. SM4,A), trajectories of treated and washed cells tend to converge with trajectory of DMSO (Fig. SM4, B) as the dose diminishes. Fig. SM5 demonstrates the Inverted Mean Average Precision (=1-mAP) computed between pairs of cells treated at 0.625μM and untreated cells in the washed part of the plate at all possible pairs of timepoints. The higher Inverted mAP demonstrates more similarity between treated and untreated cells, and Fig. SM4 shows how later timepoints of treated phenotypes land on the trajectory of untreated cells with a lag, potentially owing to confluence.

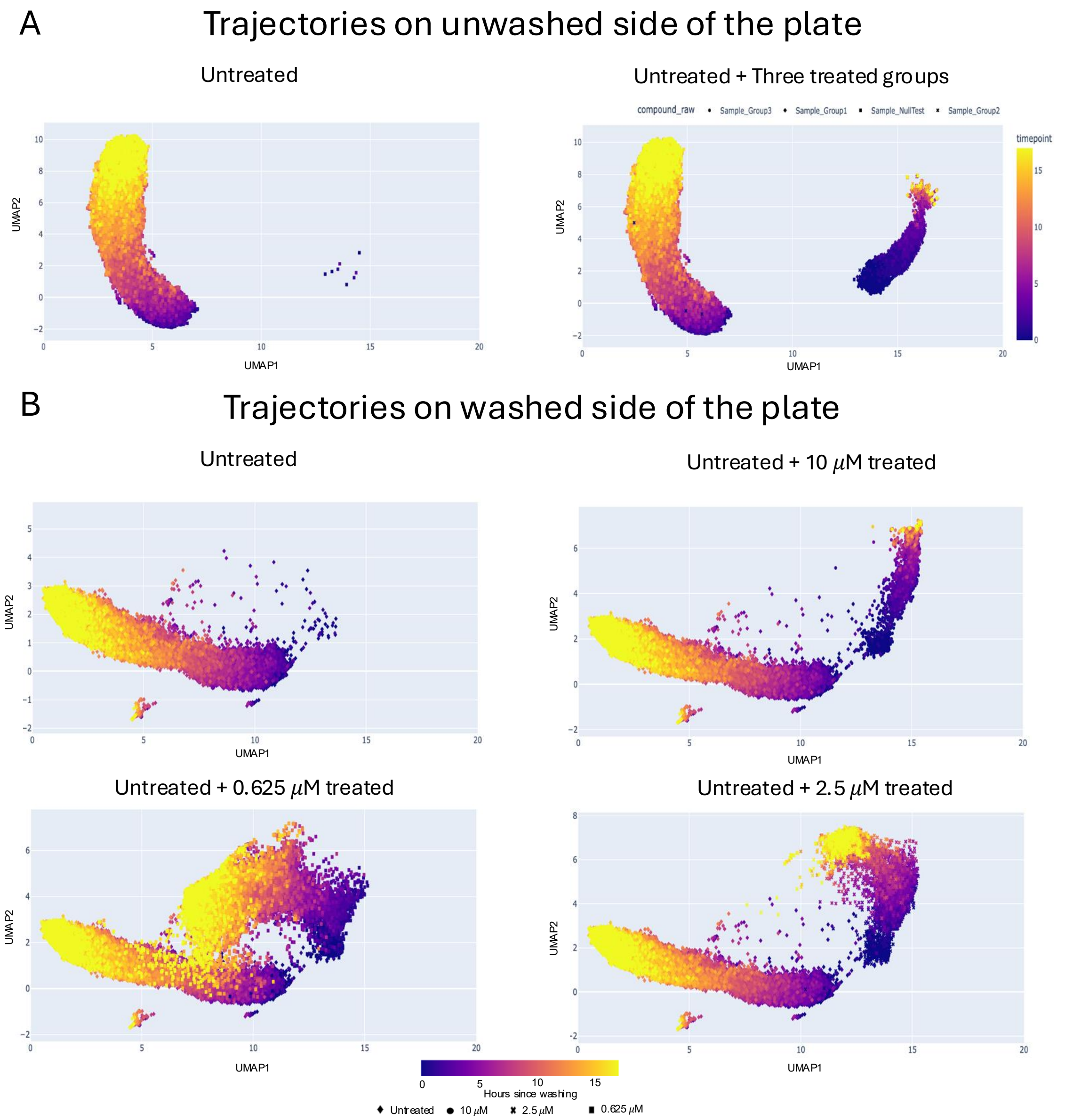

Fig. SM4. A. Trajectory of two-dimensional UMAP projections of untreated cells on the unwashed part of the plate (left) and both untreated and all three treated groups on the unwashed part of of the plate (right). Low amount of samples at later timepoints is a consequence of attrition due to low confluency (cell death following Bortezomib treatment). B. Trajectories of untreated samples and combinations of untreated samples and samples treated with different doses on the washed part of the plate. The lower the dose, the stronger is the pattern of recovery after drug withdrawal – embeddings of treated cells drifting closer to the DMSO trajectory.

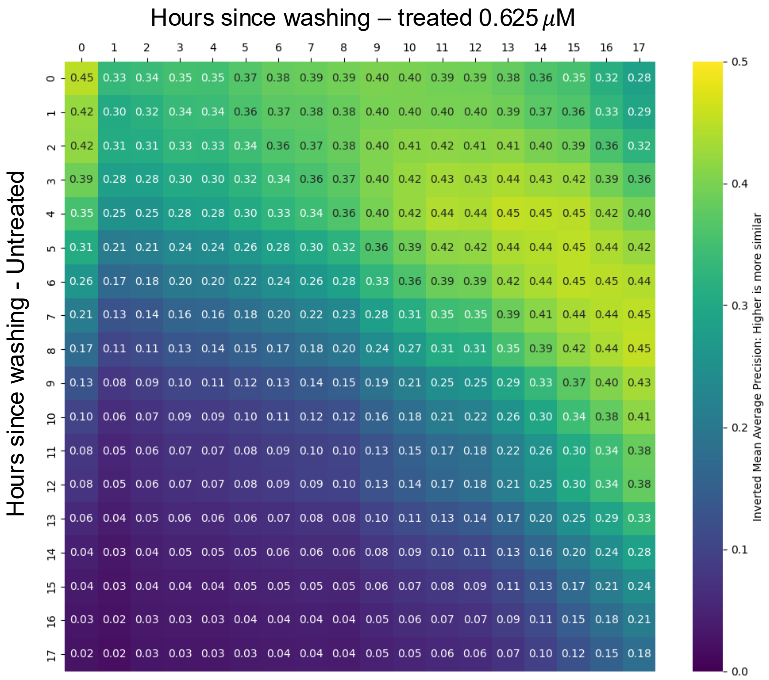

Fig. SM5. Similarity between pair of treated and washed (0.625 μM) and untreated cells, measured as Inverted mAP (1-mAP). At later timepoints treated cells partly recover to untreated phenotype with a time lag. Time lag likely is explained by cell confluency – growth of untreated cells is uninhibited, while growth of treated cells after drug withdrawal is influenced by the original drug perturbation

REFERENCES

1. Singh, S., Bray, M.-A., Jones, T. R. & Carpenter, A. E. Pipeline for illumination correction of images for high-throughput microscopy. *J. Microsc.* **256**, 231–236 (2014).
2. Čepa, M. Segmentation of Total Cell Area in Brightfield Microscopy Images. *Methods Protoc.* **1**, 43 (2018).
3. McNemar, Q. Note on the sampling error of the difference between correlated proportions or percentages. *Psychometrika* **12**, 153–157 (1947).
